# Shedding Light on a New Treatment for Diabetic Wound Healing: A Review on Phototherapy

**DOI:** 10.1155/2014/398412

**Published:** 2014-01-06

**Authors:** Nicolette N. Houreld

**Affiliations:** Laser Research Center, Faculty of Health Sciences, University of Johannesburg, P.O. Box 17011, Doornfontein 2028, South Africa

## Abstract

Impaired wound healing is a common complication associated with diabetes with complex pathophysiological underlying mechanisms and often necessitates amputation. With the advancement in laser technology, irradiation of these wounds with low-intensity laser irradiation (LILI) or phototherapy, has shown a vast improvement in wound healing. At the correct laser parameters, LILI has shown to increase migration, viability, and proliferation of diabetic cells *in vitro*; there is a stimulatory effect on the mitochondria with a resulting increase in adenosine triphosphate (ATP). In addition, LILI also has an anti-inflammatory and protective effect on these cells. In light of the ever present threat of diabetic foot ulcers, infection, and amputation, new improved therapies and the fortification of wound healing research deserves better prioritization. In this review we look at the complications associated with diabetic wound healing and the effect of laser irradiation both *in vitro* and *in vivo* in diabetic wound healing.

## 1. Introduction

### 1.1. Diabetes and Wound Healing

Diabetes Mellitus (DM) is a chronic metabolic disorder due to an absence of, insufficiency in, or resistance to insulin. Complications arise as a result of elevated glucose levels and protein glycation and include cardiovascular disease, retinopathy, nephropathy, angiopathy, and neuropathy. Patients are more likely to have foot problems due to blood vessel and nerve damage and often suffer from sensory loss. Small sores can develop on the feet and often go unnoticed. These later develop into deeper ulcers which become slow to heal, and further complications such as infection arise which often necessitate amputation due to the spread of infection to the underlying tissue and bone. It is estimated that 15–25% of patients will develop foot ulcers [1, 2], of which 6% of these will require hospitalization to treat these ulcers [3]. Around 15–20% of these patients will require lower limb amputation [1] and around 50% of all non traumatic amputations are as a result of DM [4]. To further highlight the seriousness of diabetes associated lower-limb amputations, the 5-year mortality rate following amputation stands at 39–80% [5].

To control the development of lower-limb ulcers, patients are required to check their feet daily, wear the correct footwear, and regularly visit their health care provider. It is estimated that more than 200 million people worldwide suffer from DM, and in 2004, 3.4 million people died as a result of DM [6]. DM and its associated complications impact heavily on the patient, their family, health care departments, and countries. The treatment of chronic wounds is becoming more of a burden due to the increase in health care costs, an aging population, and an increase in the incidence of diabetes [7].

**Figure 1:**
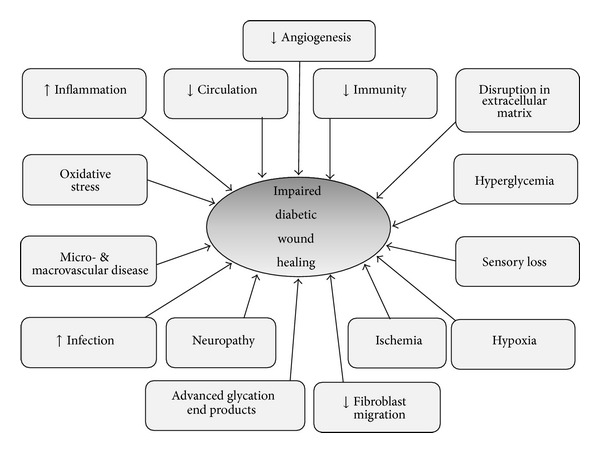
Some of the underlying pathogenesis of impaired diabetic wound healing.

Despite the huge amount of research into the underlying pathogenesis of impaired diabetic wound healing, there is still no clear answer and it appears to be a net result of micro- and macrovascular disease [8] and inadequate angiogenesis (Figure 1). Neuropathy and sensory loss have also been recognized as a major cause of prolonged healing in diabetic patients. In addition, advanced glycation end products also contribute to the pathogenesis [8], and hyperglycemia adds to the oxidative stress.

There is a decrease in wound strength, reduced angiogenesis, and poor wound contraction [8, 9]. In DM, there is a disruption in clot formation and the inflammatory phase is dysregulated [8, 10], often with a prolonged and excessive inflammatory response. Hypoxia is associated with diabetic wounds and further amplifies the inflammatory response [11]. Formation of the extracellular matrix (ECM) is a crucial step in wound healing and provides structural integrity to tissue. In diabetic wound healing, there is a malformation of the ECM due to the disruption in ECM-growth factor interactions and impaired migration and proliferation of fibroblasts [9]. Collagen is an important component of the ECM and is synthesized and maintained by a balance between matrix synthesis and degradation. In DM, there is an imbalance between matrix degrading enzymes, matrix metalloproteases (MMPs), their inhibitors, and tissue inhibitor metalloproteinases (TIMPs). The loss of collagen which is associated with diabetes can be due to decreased levels of its synthesis, enhanced metabolism, or a combination of both [12]. Nonhealing diabetic foot wounds display elevated MMP activity, with a 30- to 60-fold increase in MMP-2 and MMP-9 [11, 13]. Dysregulated cellular functions also play a part, such as defective T cell immunity, leukocyte chemotaxis, phagocytosis, and bactericidal capacity [11].

Infection of diabetic ulcers remains a real problem. It can become life threatening and is one of the most common causes of lower-limb amputation and appropriate treatment is essential [14]. Wounds are commonly infected with *Pseudomonas aeruginosa* and *Staphylococcus* [11]. Infection can spread from one ulcer to another as the foot has several intercommunicating compartments, and combined with sensory loss patients can continue walking on these infected ulcers further facilitating their spread [1]. Ischemia complicates matters further by reducing defense mechanisms. Administration of antibiotics has its own complications especially with the emergence of antibiotic resistant bacteria, poor arterial supply which affects antibiotic delivery, correct duration of treatment, and toxicity and allergy to patients.

### 1.2. Treatment of Diabetic Wounds

Management of the diabetic foot is multidisciplinary and can become problematic. Treatment is both local (treating of the diabetic foot) and systemic (glycemic control). Treatment of the diabetic foot is extensive and can encompass mechanical and surgical debridement, management of the wound base, antibiotic therapy to treat infection, revascularization, prophylactic foot surgery, mechanical off-loading, accommodative orthotics, and an alteration of footwear [2, 14]. Hyperbaric oxygen therapy (HBOT), which entails delivering 100% oxygen at pressures above one atmosphere, increases the amount of oxygen dissolved in the blood and has been used to treat a variety of wounds [12, 15]. HBOT has been used as an adjunctive treatment for diabetic foot ulcers; however its evidence of efficiency is limited [12]. Kaya and colleagues [16] treated 184 patients with diabetic foot ulcers with HBOT. Sixty-three percent of patients responded to treatment, while 17% showed no improvement and 21% underwent amputations. Complications associated with HBOT are not common but can include claustrophobia, ear, sinus, or lung damage, temporary worsening of short sightedness, and oxygen poisoning [12].

A number of clinical applications have been found for lasers in a variety of medical specialities and have been used in dentistry, dermatology, osteology, physiotherapy, acupuncture, surgery, photodynamic cancer therapy, and chiropractic and veterinary science. Lasers have also been used in the treatment of chronic wounds, including diabetic ulcers.

## 2. Phototherapy

Phototherapy, also known as photobiomodulation, low-level laser therapy (LLLT), involves the application of light (often laser light of a specific wavelength or a light emitting diode, LED) to stimulate cellular processes.

The effects of phototherapy are chemical and not thermal. Energy which is delivered to cells produces insignificant and minimal temperature changes, typically in the range of 0.1–0.5°C [17]. Cellular responses are the result of changes in photoacceptor molecules, or chromophores. Photoacceptors take part in cellular metabolism and are not connected to a light response, such as chlorophyll which is a photoreceptor [18]. Once the photon energy is absorbed, the photoacceptor assumes an electronically excited state [18], which in turn stimulates cellular metabolism [19, 20] by activating or deactivating enzymes which alter other macromolecules such as DNA and RNA [21, 22]. The energy which is absorbed by the photoacceptor can be transferred to other molecules causing chemical reactions in the surrounding tissue; this then gives rise to observable effects at a biological level [18, 23]. Photon energy is absorbed by the chromophores and there is an increase in adenosine triphosphate (ATP) [24, 25] and cell membrane permeability, which leads to activation of secondary messengers which in turn activate a cascade of intracellular signals [26]. There is also an increase in mitochondrial membrane potential and proton gradient [25].

**Figure 2:**
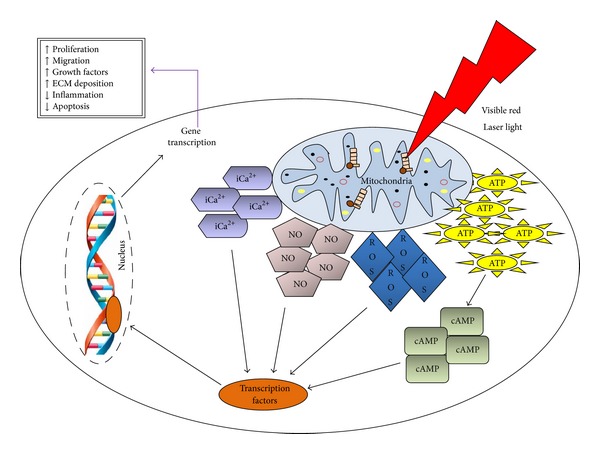
Laser light is absorbed by chromophores in the cell, mitochondria in the case of visible red light. This leads to an increase in adenosine triphosphate (ATP), reactive oxygen species (ROS), nitric oxide (NO), and intracellular calcium (iCa^2+^). There is an activation of transcription factors which get translocated to the nucleus and activate gene transcription. This leads to increased cell survival and wound healing.

The exact mechanisms of action following laser irradiation are not well understood, and a number of theories exist, the most studied and best understood being that of cytochrome-c oxidase (cyt *a*/*a*_3_), the terminal enzyme in the eukaryotic mitochondrial respiratory chain (complex IV). Cytochrome c oxidase facilitates the transfer of electrons to molecular oxygen. The end product of this complex is the production of ATP. Cytochrome c oxidase has two heme moieties (heme *a* and heme *a*_3_) and two redox-active copper sites (Cu_A_ and Cu_B_), and these are the possible absorbing chromophores for visible red and near infrared (NIR) light [18, 27, 28]. When photon energy is absorbed by cytochrome c oxidase, there is a change in the mitochondrial redox state and/or pumping of protons across the inner mitochondrial membrane [25] and an increase in ATP synthesis. There is also an increase in intracellular calcium ([Ca^2+^]i) which stimulates DNA and RNA synthesis [23]. It has been speculated by Karu [27] that photoirradiation may intensify the transfer of electrons within cytochrome c oxidase by making more electrons available. An increase in the transfer of electrons and protons accelerates oxidative metabolism which ultimately leads to increased ATP [25]. Photoirradiation causes the reduction or oxidation of cytochrome c oxidase and is dependent on the initial redox status of the enzyme at the time of irradiation [28]. Silveira and colleagues [25, 29] showed that LILI produced an increase in mitochondrial complexes I, II, III, and IV, as well as succinate dehydrogenase. Hu and colleagues [30] also found an increase in cytochrome c oxidase activity and concluded there was a cascade of reactions which altered cellular homeostasis. Houreld et al., [31] showed that irradiation of isolated mitochondria resulted in an increase in cytochrome c oxidase (complex IV) activity. There is also an increase in the concentration of active mitochondria in irradiated cells. Both effects lead to an increase in ATP. The effect of laser irradiation on the mitochondria at a transcriptional level was also investigated, and there is evidence that that there is an upregulation of genes involved in complexes I, IV, and V [32] (Figure 2).

A second possibility is the localized transient heating of the photoacceptor which may cause structural changes and trigger mechanisms such as activation or inhibition of enzymes [28]. Another theory is the release of nitric oxide (NO) from reduced cytochrome c oxidase which reverses the signalling consequences of excessive NO binding [28, 33, 34], as NO in very low concentrations inhibits cytochrome c oxidase by competing with oxygen [33, 34].

### 2.1. *In Vitro* Effects of Photoirradiation

A number of studies, on various cell types, have shown positive effects of photoirradiation. Studies have been conducted on stem cells [35–38], keratinocytes [39, 40], mast cells [41, 42], fibroblasts [43–48], smooth muscle cells [49], osteoblasts [50, 51], and schwann cells [52] to name but a few.

Impaired diabetic wound healing has been associated with impaired cellular function, and there is a decrease in cellular migration, proliferation, NO synthesis, growth factors, and collagen synthesis. There is also an increase in proteinases that degrade the extracellular matrix and collagen (MMPs) and cells appear to be stuck in the inflammatory phase of wound healing. The increase in oxidative stress also leads to increased cell death. Laser irradiation *in vitro* has shown that these cells respond in a favourable fashion, even irradiation of diabetic cells (Table 1). There is an increase in cellular migration [45, 46], proliferation [44, 46, 53–56], viability [44, 46], collagen production [45, 49, 57–59], ATP [31], mitochondria concentration [31], cytochrome c oxidase activity [31], NO [60], growth factors [57, 58, 61, 62], and gene regulation [32]. There is also a decrease in MMPs [49], apoptosis [46, 55, 63] and proinflammatory cytokines [46].

Irradiation of hypoxic cells has also shown favourable effects, with an increase in ATP and cyclic adenosine monophosphate (cAMP) [64], proliferation [46, 51], viability [46], transforming growth factor-*β*1 (TGF-*β*1) [51], intracellular Ca^2+^ [64] and mitochondrial membrane potential [64], and a decrease in apoptosis and the pro-inflammatory cytokine tumour necrosis factor alpha (TNF-*α*) [46]. Irradiation of hypoxic/ischemic cells resulted in reduced ROS, which results in increased angiogenesis [65]. Laser irradiation restores homeostasis of injured and stressed cells, resulting in improved repair and wound healing.

Not all studies have shown positive effects. Pereira et al. [54] and Marques et al. [66] showed that laser irradiation of fibroblast cells had no effect on the synthesis of procollagen. In fact there were ultrastructural changes to the endoplasmic reticulum which may have resulted in a disruption in protein synthesis [54]. Damante et al. [62] demonstrated that irradiation at 660 nm had no effect on basic fibroblast growth factor (bFGF). Interestingly, irradiation of the same cells at 780 nm significantly increased bFGF. Hakki and Bozkurt [57] irradiated human gingival fibroblasts by different laser parameters and found no increase in proliferation at each of the parameters used. However, they did find an increase in the transcription of various growth factors, namely, insulin-like growth factor (IGF), vascular endothelial growth factor (VEGF), and transforming growth factor-beta (TGF-*β*). Irradiation of hypertrophic scar-derived fibroblasts and normal dermal fibroblasts at a wavelength of 880 nm and a fluence of 2.4 and 4 J/cm^2^ had an inhibitory effect [67]. Pereira and colleagues [68] found no benefit when they irradiated human dental pulp stem cells at 660 nm using various fluencies (0.05, 0.30, 7, and 42 J/cm^2^). Schwartz-Filho and colleagues [69] showed that irradiation at a wavelength of 685 nm with a density of 25, 77, or 130 J/cm^2^ had no effect on osteogenic cell growth or viability.

These adverse effects and difference can be explained by differences in laser parameters. The effects of laser irradiation are highly dependent on the laser parameters such as wavelength, power density, and fluence. Cells respond to LILI in a dose- and wavelength-dependent manner, and the number of exposures as well as the time between exposures plays an important role [47, 70–72]. Higher fluencies have a negative effect on cells, while too low fluences have no effect. The influence of wavelength was demonstrated by Gupta et al., [73] who demonstrated that irradiation at 635 and 810 nm had a positive effect on wound healing, while a wavelength of 730 and 980 nm had no effect. This can be explained by the absorption spectrum of chromophores which absorb light at different wavelengths.

### 2.2. *In Vivo* Effects of Photoirradiation

A limited number of clinical studies have been done on diabetic wound healing (Table 1). A reason for the small number of randomized trials may be due to ethical issues associated with doing human clinical trials [67]. A number of studies using phototherapy in animal models have been done (Table 1).

Al-Watban [74] irradiated Sprague-Dawley rats (*n* = 893) to different wavelengths (532, 633, 810, 980, 10,600 nm, and 510–872 nm LED cluster) and fluencies (5, 10, 20, and 30 J/cm^2^). He showed that phototherapy at a wavelength of 633 nm accelerated healing and was the best for alleviating diabetic wounds and burn healing. It was suggested that phototherapy with 633 nm should be given three times a week at a fluence of 4.71 J/cm^2^ per dose for the treatment of diabetic burn wounds or 2.35 J/cm^2^ for diabetic wounds. Chung et al. [75] treated full-thickness circular wounds (5 mm) in diabetic mice (type 2 diabetes) with a 660 nm laser to various fluencies (0–10 J/cm^2^) for seven consecutive days. Wounds were splintered to minimize contraction; the main process of healing would thus be epithelization and granulation. On day 14 mice were euthanized and the wound excised. At a fluence between 3.7 and 10 J/cm^2^, splinted irradiated wounds responded better and healed quicker than nonirradiated splintered wounds. Santos et al. [76] irradiated cutaneous flaps with poor circulation in diabetic Wistar rats either with 680 or 790 nm (2.5 J/cm^2^ per point). It was shown that angiogenesis was increased in irradiated rats compared to control, non-irradiated rats, more so when a wavelength of 790 nm was used.

Irradiation of diabetic male Wistar rats reduced the expression of MMP and accelerated collagen production [77]. Firat and colleagues [78] irradiated full thickness wounds in diabetic male Wistar rats with a 940 nm diode laser (10 J/cm^2^). Histopathological analysis revealed that there was a decrease in inflammatory cells and an increase in collagen and vascularization. Blood tests showed that there was a decrease in oxidative stress. LILI has been found to promote healing in both soft and hard tissue. Irradiation at 980 nm has been shown to stimulate wound healing and the formation of new bone [79]. Peplow and colleagues [84] demonstrated that the effect of irradiation is due to cellular and biochemical changes in the wound environment, rather than a hypoglycemic effect. One of the long-term complications of diabetes includes musculoskeletal abnormalities, and it is a source of disability in these patients. Laser irradiation (780 nm, 5 J/cm^2^) of cryoinjured diabetic male Wistar rats showed improved muscle repair, with enhanced reorganization of the myofibers and the perimysium and reduced fibrosis [80].

Caetano et al. [81] conducted a randomized placebo-controlled double-blind study on 20 patients with a total of 32 chronic venous ulcers. Patients were divided into three groups: in group one (placebo), ulcers were cleaned with saline and treated with 1% silver sulfadiazine (SDZ) cream and patients received a placebo phototherapy; in group two ulcers were treated similarly and patients received phototherapy (combined 660 nm and 890 nm at a fluence of 3 J/cm^2^); and in group three (controls) ulcers were treated similarly and received no phototherapy. Patients that received phototherapy responded to the laser treatment, and ulcers healed significantly faster than the control and placebo group, particularly larger ulcers. Minatel et al. [82] showed that combined irradiation with 660 and 890 nm promoted granulation and healing of diabetic ulcers that failed to respond to other forms of treatment. Kaviani et al. [83] performed a randomized study on diabetic patients with foot ulcers that would not respond to other treatments. Patients were divided into two groups: group one received conventional treatment and placebo irradiation, while group two received conventional treatment and phototherapy (685 nm, 10 J/cm^2^). The size of the ulcers in the phototherapy treated group was significantly smaller and the average time of healing was 11 weeks as opposed to 14 weeks as observed in the placebo group.

Infection in diabetic wounds is a major problem, and eradication with antibiotics proves difficult due to decreased blood flow. Laser irradiation has also been shown to inhibit bacteria. Enwemeka et al. [85] showed a dose-dependent decrease in the number of methicillin-resistant *Staphylococcus aureus* (MRSA) treated *in vitro* at a wavelength of 470 nm (blue light). Irradiation at a wavelength of 408 nm was suggested by Ankri and colleagues [86] in treating infected wounds to clear the infection, followed by irradiation at 730 nm to speed up the healing process. This is an important breakthrough as combined irradiation with visible red and blue light can potentially be used to treat infected diabetic ulcers.

## 3. Conclusion

DM is the leading cause for lower limb amputations. Current treatments are challenging, lengthy, costly, and associated with failure to heal and relapse. The patient's quality of life is affected, and a burden is placed on both patients and caregivers. There is a need to develop additional therapies to treat diabetic ulcers. Due to its stimulatory effect and no reported sideeffects, laser therapy has been used to treat chronic wounds, including diabetic ulcers. Phototherapy has been shown to be beneficial in treating diabetic ulcers which are unresponsive to conventional treatments. This has led to an improvement in the quality of patient's lives. By studying the effects of LILI *in vitro*, the underlying mechanisms are being identified. The number of clinical studies in DM is limited, and there is methodological heterogeneity which explains the varied results seen. Better designed, well-controlled, randomized, and double-blind studies are needed for this type of therapy to become accepted and used as an adjuvant therapy for the treatment of diabetic ulcers. Phototherapy can be an important tool in speeding up the healing process as well as alleviating pain and inflammation. There is also a need to inform clinicians and other health care providers of the beneficial effects of phototherapy.

## Figures and Tables

**Table 1:** Summary of *in vitro* and *in vivo* studies done on various cell types and animal models, respectively, using low level laser irradiation (LILI).

Species/cell type	Study design	Outcomes	Author reference
*In vitro* studies			
Diabetic wounded human skin fibroblasts	Cells were irradiated at 660 nm with 5 J/cm^2^ and incubated for 48 or 72 h. Control cells received no laser irradiation.	Irradiation resulted in increased cellular migration, viability, proliferation, and collagen production.	Ayuk et al. [45]

Diabetic wounded and hypoxic human skin fibroblast cells (WS1)	Cells were irradiated at 636 nm with 5 J/cm^2^ and incubated for 1 or 24 h. Control cells received no laser irradiation.	Irradiated diabetic wounded cells showed increased cellular migration, viability, and proliferation and a decrease in apoptosis (caspase 3/7) and proinflammatory cytokine interleukin (IL)-1*β*. Nuclear factor kappa B (NF-*κ*B) also translocated into the nucleus. Irradiated hypoxic cells regained their normal morphology and showed an increase in cellular viability, proliferation, and IL-6 and decreased apoptosis (caspase 3/7) and proinflammatory cytokine *tumor necrosis factor* (TNF)-*α*. NF-*κ*B also translocated into the nucleus.	Sekhejane et al. [46]

Human skin fibroblasts (HSFs)	Cells were cultured in physiologic glucose (5.5 mM/L) or high glucose concentration (11.1 and 15 mM/L) and irradiated at 632.8 nm with 0.5, 1, and 2 J/cm^2^ on 3 consecutive days.	Densities of 0.5 and 1 J/cm^2^ had stimulatory effects on the viability and proliferation rate of HSFs cultured in physiologic glucose. Densities of 0.5, 1, and 2 J/cm^2^ had stimulatory effects on the proliferation rate of HSFs cultured in high glucose concentrations.	Esmaeelinejad et al. [44]

Diabetic wounded skin fibroblast cells (WS1)	Cells were irradiated at 632.8 nm with 5 or 16 J/cm^2^. Control cells received no laser irradiation.	Cells irradiated at 5 J/cm^2^ showed increased cellular migration and proliferation, while cells irradiated at 16 J/cm^2^ showed decreased cellular migration and proliferation.	Houreld and Abrahamse [53]

NIH 3T3 fibroblast cells	For proliferation studies, cells were grown in 2.5% foetal bovine serum (FBS) and irradiated at 904 nm. Cells received two applications (6 h interval) of 2 J/cm^2^ each (4 J/cm^2^ total); 1 J/cm^2^ and then 2 J/cm^2^ (3 J/cm^2^ total); 2 J/cm^2^ and then 3 J/cm^2^ (5 J/cm^2^ total). Control cells received no laser irradiation. Cells were incubated for 2, 4, 5, and 6 days. For procollagen studies, cells were grown in 2.5% FBS and irradiated at 904 nm, 3 J/cm^2^ and incubated for 4 days.	Cells irradiated with 3 and 4 J/cm^2^ showed increased cellular proliferation. No significant increase in procollagen was seen in any of the irradiated cells.	Pereira et al. [54]

Murine fibroblast 3T3 cells and primary human keloid fibroblast cell cultures	Cells were irradiated at 660 nm for 3 consecutive days (24, 48, and 72 h) with 3 or 21 J. For the MTT assay (proliferation), a power density of 0.26 W/cm^2 ^was used, while 0.63 W/cm^2^ was used for viability assays (Trypan blue).	A dose of 3 J stimulated proliferation, while 21 J inhibited proliferation of human keloid fibroblast cells. Laser irradiation is affected by the physiological state of the cells; high-metabolic rate and short-cell-cycle 3T3 cells were not responsive to LILI. A dose of 3 J reduced cell death but did not stimulate cell cycle. A dose of 21 J had negative effects on the cells, as it increased cell death and inhibited cell proliferation.	Frigo et al. [55]

HIG-82 rabbit synovial fibroblasts	Cells were synchronized at G1 by serum starvation (0.2% FBS for 24 h) and irradiated at 660 nm with 1.2, 4.8, or 7.2 J/cm^2^ and cultured for another 24 h. Control cells received no laser irradiation.	Cellular proliferation was significantly stimulated at 4.8 and 7.2 J/cm^2^, while no effect was observed at 1.2 J/cm^2^. The proportion of cells at S phase in the laser irradiation group (4.8 J/cm^2^) was significantly higher; thus LILI enhances cell cycle progression and as it promotes synovial fibroblast proliferation.	Taniguchi et al. [56]

Porcine primary aortic smooth muscle cells (SMCs)	Cells were irradiated at 780 nm with 1 or 2 J/cm^2^. Cells were incubated for different time periods depending on the assay.	LILI stimulated porcine aortic SMC proliferation, increased collagen synthesis, modulated activity and expression of matrix metalloproteinase (MMP)-2, gene expression of MMP-1, and tissue inhibitor of metalloproteinases (TIMP)-2, and inhibited gene expression of proinflammatory cytokine IL-1*β*.	Gavish et al. [49]

Primary human gingival fibroblasts (GF)	Cells were irradiated at 780 nm with different settings used in dentistry: power: 2 W, pulse interval: 1 ms, pulse length: 1 ms, 20 s/cm^2^, 20 J/cm^2 ^(infected pocket setting); power: 1.5 W, pulse interval: 20 ms, pulse length: 20 ms, 20 s/cm^2^, 15 J/cm^2 ^ (Perio pocket setting); power: 0.3 W in continuous wave, 20 s/cm^2^, 6 J/cm^2^ (biostimulation setting).	No significant difference in proliferation was observed in the different laser applications when compared to the control group. Significantly increased insulin-like growth factor (IGF) and vascular endothelial growth factor (VEGF) mRNA was observed in all irradiated groups. A significant increase in collagen type I mRNA expression was noted in only the biostimulation setting.	Hakki and Bozkurt [57]

Human foreskin fibroblast HS68 cells	Cells were grown in 1% FBS for 24 h and then irradiated with a light emitting diode (LED) array (630 nm) with 1 or 2 J. Cells were incubated for 1 or 3 days. Control cells received no laser irradiation.	A dose of 1 J induced a significant increase in viability. Irradiation increased the mRNA expression level of type I collagen and also affected basic fibroblast growth factor (bFGF) secretion levels.	Huang et al. [58]

Human dermal fibroblasts	LED array populated with 590 and 870 nm LEDs. The ratios of visible to infrared (IR) light were decreased (in the case of visible) and increased (in the case of IR) in series of 25% increments from no IR to fully IR. Cells were incubated for 24 h.	Photomodulation with a 590/870 nm LED array in different ratios has an effect on gene expression profiles and is effective for altering gene expression, collagen synthesis, and reduction of MMP-1 expression.	McDaniel et al. [59]

Human gingival fibroblasts, FMM1 cells	Cells were irradiated at 904 nm with 3 J/cm^2^ and incubated for 3 days. Control cells received no laser irradiation.	Irradiation produced no difference in the amount of procollagen between groups, and the amount of type I collagen as well as the total protein content was significantly smaller in control cultures. There were also ultrastructural changes in cytoplasmic organelles, especially the mitochondria and rough endoplasmic reticulum.	Marques et al. [66]

Diabetic and ischemic skin fibroblast cells (WS1)	Whole cells or isolated mitochondria were irradiated at 660 nm with 5 or 15 J/cm^2^. Control cells received no laser irradiation.	Irradiation of mitochondria with 15 J/cm^2^ resulted in increased adenosine triphosphate (ATP) production, a higher accumulation of activated mitochondria in diabetic cells, an increase in complex IV activity, and a decrease in complex III activity. There was an increase in complex IV activity in mitochondria and a higher accumulation of activated mitochondria in diabetic cells irradiated with 5 J/cm^2^. Irradiated ischemic cells showed no significant differences compared to their nonirradiated control.	Houreld et al. [31]

Diabetic wounded skin fibroblast cells (WS1)	Cells were irradiated at 830 nm with 5 J/cm^2^. Control cells received no laser irradiation.Cells were incubated for 15 min, 1, 24, or 48 h.	Irradiation resulted in increased cellular proliferation (24 and 48 h), nitric oxide (15 min), and reactive oxygen species (15 min) and decreased apoptosis (24 h), TNF-*α* (1 and 24 h), and IL-1*β* (24 h).	Houreld et al. [60]

Primary human gingival fibroblasts (hGF)	Cells irradiated at 685 nm with 2 J/cm^2^ and incubated for 24 h. Two study groups, namely, cells which were irradiated once (single-dose group) and cells which were irradiated twice with 24 h interval (double dose). Control cells received no laser irradiation.	Cells in the single-dose group showed a significant increase in proliferation and growth factors bFGF and IGF-1, with no change in IGF-binding protein (IGFBP)3. Cells in the double dose group showed a significant increase in proliferation and growth factors bFGF, IGF-1, and IGFBP3.	Saygun et al. [61]

Human gingival fibroblast cell line (FGH)	Cells were grown in 1% FBS for 24 h and then irradiated in media containing 10% FBS. Cells irradiated twice at 660 or 780 nm with 3 or 5 J/cm^2^ with 6 h between irradiations.	There was no significant difference in the expression of keratinocyte growth factor (KGF), while bFGF was significantly increased in cells irradiated at 660 nm (no difference at 780 nm).	Damante et al. [62]

Wounded, diabetic wounded, and ischemic skin fibroblast cells (WS1)	Cells were irradiated at 660 nm with 5 J/cm^2^. Control cells received no laser irradiation. Cells were incubated for 30 min.	Irradiation upregulated the expression of mitochondrial genes COX6B2 (complex IV), COX6C (complex IV), and PPA1 (complex V) in diabetic wounded cells and ATP4B (complex V) and ATP5G2 (complex V) in ischemic cells. COX6C (complex IV), ATP5F1 (complex V), NDUFA11 (complex I), and NDUFS7 (complex I) were upregulated in wounded cells.	Masha et al. [32]

Isolated mouse embryonic fibroblasts	Cells irradiated at 810 nm with 0.003, 0.03, 0.3, 3, or 30 J/cm^2^. Control cells received no laser irradiation.	A dose of 0.3, 3, and 30 J/cm^2^ produced an increase in reactive oxygen species (ROS). No increase in ATP was seen with 0.003 J/cm^2^, a small increase was seen at 0.03 J/cm^2^ and a large increase was seen with fluencies of 0.3, 3, and 30 J/cm^2^. A dose of 0.3 J/cm^2^ increased NF-*κ*B 1 h after irradiation. Activation of NF-*κ*B is mediated via ROS generation.	Chen et al. [63]

*In vivo studies*			
Rat, Sprague-Dawley, diabetic (streptozotocin induced), and nondiabetic	Full-thickness wound (102.5 ± 9 mm^2^) or a burn 1 (48 ± 12.5 mm^2^) was made on each rat. Rats were irradiated with various wavelengths (532, 633, 810, 980, and 10,600 nm) and polychromatic LED clusters (510–543, 594–599, 626–639, 640–670, and 842–872 nm) with a dose of 5, 10, 20, or 30 J/cm^2^ three times per week.	The best effects on wound and burn healing were exhibited with a laser with a wavelength of 633 nm. Based on the results, phototherapy at 633 nm, 4.71 J/cm^2^, 3 times/week is recommended for diabetic burn wounds, and phototherapy at 633 nm, 2.35 J/cm^2^, 3 times/week for diabetic wounds is recommended for human clinical trials.	Al-Watban [74]

Mice, diabetic (BKS.Cg-m+/+Lepr_ _^db^/J), male and female	A full-thickness circular wound was made on the left flank in each mouse using a sterile 5 mm diameter skin punch, and the wound extended down to the fascial layer over the abdominal musculature. Wounds were irradiated at 660 nm, with 0, 0.8, 1.6, or 3.2 J/day. Mice were euthanized on day 14.	Irradiation of splintered wounds at 660 nm with 1.6 J/day (3.7–5.0 J/cm^2^/day) for 7 days was shown to cause the maximal stimulation of healing on day 14. Wounds healed mainly by reepithelization and granulation tissue formation.	Chung et al. [75]

Rats, Wistar, diabetic (streptozotocin induced), and male	A 2 × 8 cm cutaneous flap was raised on the dorsum of each animal. A plastic sheet was introduced between the flap and the bed to impair blood supply, and the flap was then sutured. Rats were treated transcutaneously every other day with 680 or 790 nm, on 16 contact points at the wound margin (2.5 J/cm^2^/point; total of 40 J/cm^2^). Rats were euthanized on day 8.	The results suggest that the best responses of the flaps were observed on irradiated subjects, in particular those treated with 790 nm. There was increased angiogenesis, reduced tissue necrosis and inflammation, and increased fibroblastic proliferation.	Santos et al. [76]

Rats, Wistar, diabetic (streptozotocin induced), and male	Rats were divided into 4 groups: control (untreated, nondiabetic); laser (laser treated, nondiabetic); diabetic (diabetic rats, nonlaser treated); and diabetic + laser (diabetic rats laser treated). Scars were irradiated once at 660 nm with 4 J/cm^2^, and rats were euthanized 24 h after irradiation.	In untreated diabetic rats there was increased MMP-2 and MMP-9 expression compared to untreated nondiabetic rats. Irradiation of diabetic rats significantly reduced MMP-2 and MMP-9 expression compared to untreated diabetic rats, and there was also increased production of collagen.	Aparecida et al. [77]

Rats, Wistar, diabetic (streptozotocin induced), male	Full-thickness wounds were made in the hard palates using a 3 mm biopsy punch. Rats were divided into 2 groups: control group (nonirradiated) and experimental group (irradiated). Wounds were irradiated at 940 nm with 10 J/cm^2^ after surgery and on days 2, 4, and 6 after surgery. Rats were euthanized on days 7, 14, and 21. Irradiation resulted in decreased numbers of inflammatory cells and increased mitotic activity of fibroblasts, collagen synthesis, and vascularization. Oxidative status was also significantly decreased on day 21.	Decreased inflammatory cells, and oxidative stress and increased collagen and vascularization	Firat et al. [78]

Rat, Sprague-Dawley, normal or diabetic (streptozotocin induced), male	Left and right maxillary first molars were extracted, and extraction sockets on the left were not irradiated, while the right ones were irradiated at 980 nm with 13.95 J/cm^2^. Rats were euthanized 3, 5, 7, or 14 days after extraction.	Irradiation promoted new bone formation. In normal rats, osteoblasts and osteoid tissue were observed at day 5, which was earlier than in the control group, and new bone reached the top of the extraction socket at day 14. In diabetic irradiated rats, less infiltration of inflammatory cells and blood clots were observed at day 3, and more new bone formed at days 7 and 14 than in the nonirradiated diabetic group. Laser irradiation stimulated the differentiation of osteoblasts and increased the expression of collagen type I and osteocalcin mRNA.	Park and Kang [79]

Rats, Wistar, diabetic (streptozotocin induced) male	Rats were divided into 7 groups: control (normoglycemic, no injury), diabetic (no injury), sham (Normoglycemic, sham irradiated), diabetic sham, nondiabetic cryoinjured submitted to LLLT, diabetic cryoinjured submitted to LLLT, and diabetic cryoinjured nontreated. Cryoinjury was carried out on the left posterior leg: the muscle fascia was carefully removed, and the tibialis anterior muscle was surgically exposed and cryoinjured for 10 s with a cooled (in liquid nitrogen) round 3 mm metal probe. After the frozen muscle had thawed, the procedure was repeated on the same area for another 10 s. Surgical wounds were closed with sutures and rats were allowed recovering. Two hours after injury, the muscle was irradiated at 780 nm with 5 J/cm^2^ to 8 points within the area (energy per point was 0.2 J, totalizing 1.6 J per treatment). Irradiations were performed daily (24 h interval). Rats that were euthanized on day 7 received 6 treatments, while rats euthanized on day 14 received 13 treatments.	Diabetic animals that received LLLT exhibited morphological aspects of skeletal muscle healing similar to those found in the normoglycemic animals having received LLLT, with the organization of immature fibers in the collagen meshwork. The diabetic sham irradiated group exhibited fibrosis. Thus, LLLT can help avoid fibrosis and reduce muscle atrophy	França et al. [80]

Double-blind, randomized, placebo-controlled study. Twenty patients with 32 chronic lower extremity venous ulcers	Inclusion criteria included the following: ulcer in the lower extremity, (2) ulcers larger than 1.0 cm^2^, (3) ulcer duration >6 wk, (4) presence of classical signs of venous insufficiency such as edema, varicosities, lipodermatosclerosis, eczema, and elephantiasis nostra, (1) and (5) controlled systemic arterial hypertension (diastolic arterial pressure <95 mm Hg). Each group of ulcers was treated 2x/week. Ulcers were covered with 1% silver sulfadiazine (SDZ) cream, dressed, and then bandaged. Group 1 received placebo phototherapy; group 2 were irradiated at 660 and 890 nm (LEDs) 30 s per point until the entire ulcer surface was treated with the probe; and the control group 3 received standard care without phototherapy. Ulcers were treated for a maximum of 90 days.	Laser therapy increased wound healing. At all time points, light treated ulcers healed faster than the control group treated with SDZ cream dressing alone, as well as the placebo treatment group.	Caetano et al. [81]

Double-blind, randomized placebo-controlled, experimental design, 14 patients with 23 chronic diabetic leg ulcers	Inclusion criteria: (1) diagnosis of type II diabetes independent of glycemic control with neuropathic or mixed (venous and arterial) ulcers, (2) ulcer located on the lower extremity, (3) ulcer present for a minimum of 4 weeks during which it has been either stable or worsening, (4) willingness to participate in the study and commitment to the follow-up protocol, and (5) signed written consent. Ulcers were cleaned with 0.9% physiological saline and dried before phototherapy was applied twice per week for a maximum of 90 days. Ulcers were dressed with 1% silver sulfadiazine cream covered with gauze and bandaged. Ulcers in the irradiated group were treated with 660 and 890 nm probes (LEDs) 30 s per point until the entire ulcer surface was treated.	Laser irradiation using a combination of 660 and 890 nm promoted tissue granulation and rapid healing of diabetic ulcers that failed to respond to other forms of treatment.	Minatel et al. [82]

Double-blind, randomized controlled clinical trial, 23 patients with chronic diabetic ulcers	Patients having a diabetic foot ulcer for a minimum of 12 weeks with ulcer stages I and II who were capable of giving informed consent, understanding instructions, and cooperating with study protocol were enrolled. Patients were divided into laser treated and conventional therapy or conventional therapy alone (placebo group). Ulcers were treated 6x/week for two successive weeks and then every other day up to complete healing. Ulcers were treated with a 685 nm laser at a dose of 10 J/cm^2^. Patients in the placebo treatment group received sham irradiation.	Laser irradiation increased wound healing. Four weeks after beginning treatment, the size of ulcers was significantly decreased and by 20 weeks a greater number of patients in the irradiated group showed complete healing than in the placebo group, and the mean time of healing was lower.	Kaviani et al. [83]
